# Immunofluorescent visualization of mouse interneuron subtypes

**DOI:** 10.12688/f1000research.5349.2

**Published:** 2014-11-20

**Authors:** Simon Molgaard, Maj Ulrichsen, Simon Boggild, Marie-Louise Holm, Christian Vaegter, Jens Nyengaard, Simon Glerup

**Affiliations:** 1 The Lundbeck Foundation Research Center MIND, Department of Biomedicine, Aarhus University, Aarhus, 8000 C, Denmark; 2 Stereology and Electron Microscopy Laboratory, Department of Clinical institute, Aarhus University, Aarhus, 8000 C, Denmark; 3 Department of Neuroscience, Mayo Clinic, Jacksonville, FL, FL 32224, USA; 4 Danish Research Institute of Translational Neuroscience DANDRITE, Aarhus, 8000, Denmark

## Abstract

The activity of excitatory neurons is controlled by a highly diverse population of inhibitory interneurons. These cells show a high level of physiological, morphological and neurochemical heterogeneity, and play highly specific roles in neuronal circuits. In the mammalian hippocampus, these are divided into 21 different subtypes of GABAergic interneurons based on their expression of different markers, morphology and their electrophysiological properties. Ideally, all can be marked using an antibody directed against the inhibitory neurotransmitter GABA, but parvalbumin, calbindin, somatostatin, and calretinin are also commonly used as markers to narrow down the specific interneuron subtype. Here, we describe a journey to find the necessary immunological reagents for studying GABAergic interneurons of the mouse hippocampus. Based on web searches there are several hundreds of different antibodies on the market directed against these four markers. Searches in the literature databases allowed us to narrow it down to a subset of antibodies most commonly used in publications. However, in our hands the most cited ones did not work for immunofluorescence stainings of formaldehyde fixed tissue sections and cultured hippocampal neurons, and we had to immunostain our way through thirteen different commercial antibodies before finally finding a suitable antibody for each of the four markers. The antibodies were evaluated based on signal-to-noise ratios as well as if positive cells were found in layers of the hippocampus where they have previously been described. Additionally, the antibodies were also tested on sections from mouse spinal cord with similar criteria for specificity of the antibodies. Using the antibodies with a high rating on pAbmAbs, an antibody review database, stainings with high signal-to-noise ratios and location of the immunostained cells in accordance with the literature could be obtained, making these antibodies suitable choices for studying the GABAergic system.

## Introduction

Hippocampal networks are composed of a large portion of excitatory principal cells and a smaller cohort of inhibitory interneurons ^1^. Inhibitory interneurons release γ-aminobutyric acid (GABA), which is the major inhibitory neurotransmitter in the brain. Its principal action is mediated through ubiquitous fast ionotropic GABA _A_ receptors by increasing the membrane permeability to Cl ^-^ ions ^2^. This inhibitory mechanism regulates the excitability of both principal cells and GABAergic interneurons. In this way, GABA is able to efficiently control the rhythms of cortical networks ^3^, which is believed to be of critical importance for information processing ^4^ alterations in cortical network rhythms in specific brain networks that may underlie neuropsychiatric disorders, such as schizophrenia, depression and bipolar disorder, is thought to involve a defective GABA system ^5^.

Inhibitory interneurons of the dentate gyrus is a highly diverse population and early studies identified up to 21 different subtypes in this region alone ^6^. Immunostaining against GABA have shown discrepancy when compared to *in-situ* hybridization against glutamate decarboxylase, the enzyme that catalyzes the decarboxylation of glutamate to GABA, indicating that some cells may express very low levels of GABA leaving this as an insufficient choice for immunostaining ^7– 9^. These 21 subtypes can be distinguished based on axonal distribution, synaptic targets, neuropeptide or calcium-binding protein content and physiological characteristics ^10^. In order to fully characterize a subtype, all parameters must be taken into account. When immunostaining against neuropeptides or calcium-binding proteins, this is not possible, and immunostaining therefore only allows characterization of subgroups.

One such subgroup is the parvalbumin expressing interneurons. Parvalbumin-labelled cell bodies are found primarily near the granule cell layer and are most prominent at the base of the granule cell layer. However, few are also found near the border of the granule cell and molecular layers and some in the hilus as well ^10^. Although this is considered the largest group of the subgroups in the hippocampus, in the dentate gyrus these only represent around 20% of the total number of GABAergic interneurons as compared to around 40% in CA1 and CA3 ^11^.

Several distinct populations are found that express the calcium-binding protein calretinin. Most notably, calretinin is also found in mossy cells of the hilus ^12^, and such mossy cells are particular numerous in the ventral hilus. Calretinin is also found in axon terminals of mossy cells which creates a dense band of labelling in the inner third of the molecular layer ^13^.

Despite labelling of mossy cells in the hilus, some GABAergic interneurons can also be found in the hilus near the granule layer ^14^. These can often be distinguished by the more intense labelling when staining for calretinin compared to that of mossy cells.

Another subgroup is the somatostatin expressing interneurons. This subgroup comprises the largest group of GABAergic interneurons in the dentate gyrus and these are almost exclusively found within the hilus where they comprise approximately 55% of the total number of GABAergic interneurons with a slight increase from the dorsal to the ventral part of hippocampus ^15^. As almost all somatostatin positive interneurons are found within the hilus, little labelling is found within the granule cell layer, except from a large number of axons from hilar somatostatin interneurons that project through this layer ^15, 16^.

Calbindin has been found to be present in both inhibitory and excitatory neurons with a rather strong staining of granule cells in the dentate gyrus. Misplaced granule cells found in the stratum radiatum of the CA3 subfield are often mistaken for GABAergic interneurons but these are not positive for GABA ^1^. All other cells in the dentate gyrus should be considered GABAergic interneurons and generally stain for GABA ^1^. A precise percentage of calbindin interneurons is not available, but around 10–12% of total number of GABAergic interneurons is considered a close estimate ^17^. Very few calbindin positive interneurons are found in the dentate gyrus compared to the CA-regions and these are difficult to detect due to the strong staining of granule cells, but calbindin positive interneurons can be found in the stratum moleculare and hilus ^1^.

Importantly, markers of hippocampal GABAergic interneurons do not readily apply to other regions such as the spinal cord GABAergic interneurons. The inhibitory interneurons of the spinal dorsal horn use primarily GABA and/or glycine. GABAergic interneurons are primarily located in laminae I, II and III of the dorsal horn and constitute approximately 25%, 30% and 40% of rat laminae I, II and III neurons, respectively ^18, 19^. The inhibitory effect of glycine is facilitated by activation of ionotropic ligand-gated glycine receptors that mediate an influx of chloride ions ^20^ and within lamina I-III glycine immunostaining is largely restricted to GABAergic neurons ^18, 19^.

GABAergic interneurons of the spinal dorsal horn can be identified by immunostaining against, for instance, parvalbumin and the neuronal form of nitric oxide synthase (n-NOS) besides GABA and glycine. Parvalbumin is expressed by a subpopulation of spinal cord dorsal horn interneurons that co-express GABA and glycine ^21– 23^. Conversely, calretinin, somatostatin and calbindin do not co-localize with GABA in interneurons of the dorsal horn, for which reason they are thought to co-localize to excitatory interneurons ^21, 23– 25^. Thus, care should be taken when extrapolating interneuron markers from one region of the CNS to another. In the present study, we have evaluated a number of different antibodies ( Table 2) against GABAergic markers using both cultured neurons and tissue sections. All tested antibodies have previously been reported to recognize GABAergic interneurons both in peer-reviewed publications and by the manufacturers.

## Materials and methods

All experiments were approved by the Danish Animal Experiments Inspectorate under the Ministry of Justice (Permit 2011/561-119) and carried out according to institutional and national guidelines.

For a full list of reagents and chemicals, please see Table 1.

### Hippocampal section preparation and immunostaining

- Hippocampal sections. Adult C57BL/6j Bomtac (wild type (wt)) mice (Taconic), aged 8 weeks were deeply anesthetized by intraperitoneal injection of 5 mg/ml pentobarbital and perfused transcardially with cold 4% (w/v) formaldehyde (pH 7.4, Hounisen) for five minutes. The brains were hereafter removed and post-fixed in 4% (w/v) formaldehyde overnight at 4°C. The next day the brains were moved to 30% (w/v) sucrose (Merck Millipore) for cryoprotection and left at 4°C for 48 hours, moulded in Tissue-Tek ^®^ (Sakura) and stored at -20°C. Coronal hippocampal sections (10 µm) were cut at -20°C using a Leica CM1900 cryostat (using low-profile disposable blades 819 from Leica Biosystems) and the sections were afterwards stored at -20°C until use.
- Immunostaining of tissue. Antigen epitopes shielded by formaldehyde cross-linked lysine side chains were retrieved in a heat-mediated antigen retrieval step using Target Retrieval Solution (Dako), according to manufacturers’ protocol. Hereafter, the sections were washed three times in Tris-buffered saline (TBS; pH 7.4) of ten minutes intervals, and incubated in a solution of TBS containing 0.3% Triton X-100 (Applichem) and 1% bovine serum albumin (BSA; Sigma) for thirty minutes. Following a ten minute washing step in TBS, the sections were incubated with primary antibody ( Table 2) in a 50 mM Tris-based (TB) buffer solution (pH 7.4) containing 1% BSA (Sigma) at 4°C in a moisturized chamber overnight. The next day, the sections were left at room temperature (RT) for one hour, and subsequently washed three times in TBS. Sections were then incubated with secondary antibody ( Table 3) in a 50 mM TB buffer solution containing 1% BSA (Sigma) at RT for four hours. Finally, the sections were washed three times five minutes in TBS, with Hoechst (5 µg/µl, Sigma-Aldrich) being included in the last wash. The sections were hereafter mounted using Fluorescence Mounting Medium (Dako) and stored at 4°C. As negative controls of the immunostaining, simultaneous stainings were done using a similar protocol, except primary antibody was omitted. All immunostatings were tested on at least three different wild type males and repeated at least three times.

### Spinal cord section preparation and immunostaining

- Spinal cord sections. Adult C57BL/6j Bomtac (wt) mice aged 16 weeks were deeply anaesthetized using 4% isoflurane (IsoFlo ^®^ vet, Abbott) prior to decapitation and hydraulic spinal cord extrusion ^26^ using ice-cold phosphate-buffered saline (PBS; pH 7.4) as the extrusion liquid. Spinal cords were fixed in 4% (w/v) paraformaldehyde (PFA; Sigma) in PBS (pH 7.4) overnight at 4°C. The spinal cords were then cryoprotected overnight by immersion in 25% (w/v) sucrose in PBS (pH 7.4) at 4°C. Lumbar sections 2–4 of the spinal cords were isolated and embedded in TissueTek ^®^ (Sakura) prior to freezing, which was performed by lowering the tissue into dry-ice cold iso-pentane (VWR BDH Prolabo ^®^). The tissues were stored at -80°C until further use. Transverse sections of 20 μm thickness were cut at -20°C using the CryoJane ^®^ Tape-Transfer System (Leica Microsystems) on a Leica CM1900 cryostat (using low-profile disposable blades 819 from Leica Biosystems) and the sections were stored at -20°C.
- Immunostaining of tissue. This step was done similar to previously described for immunostaining of hippocampal tissue.

### Primary hippocampal neurons culture preparation and immunostaining

- Culture of primary hippocampal neurons. Postnatal day 0 (P0) C57BL/6j Bomtac (wt) mice pups were sacrificed by decapitation, brains removed and hippocampi dissected into ice cold PBS. The tissue was dissociated for thirty minutes in 20 U/mL activated papain (Worthington Biochemical Corporation). After dissociation, the tissue was washed once in DMEM (Lonza) containing 0.01 mg/mL DNaseI (Sigma) before being triturated in DMEM (Lonza) containing 0.01 mg/mL DNaseI (Sigma). After this, Neurobasal-A medium (Gibco) containing B-27 Supplement (Gibco), 2 mM GlutaMAX (Gibco), 100 μg/mL Primocin (Invivogen) and 20 μM floxuridine + 20 μM uridine (Sigma) was added to the cells and the cells were seeded on poly-D-lysine (Sigma-Aldrich) and laminin (Invitrogen) pre-coated coverslips at a density of 100.000 cells per coverslip and left for fourteen days at 37°C and 5% CO _2_, with medium change every second day, before being fixed in PBS containing 4% PFA.
- Immunostaining of cultured hippocampal neurons. Neurons fixed in 4% PFA was briefly washed in PBS prior to three consecutive washes in PBS containing 0.1% Triton X-100 of ten minute intervals. Hereafter, the cells were washed once in PBS before being incubated in PBS containing 10% FBS (Gibco) for thirty minutes at RT. After this, the cells were incubated with primary antibody ( Table 2) overnight at 4°C. The next day, the immunostaining were left at RT for one hour before continuing the immunostaining protocol. Hereafter, the cells were washed three times five minutes in PBS containing 0.1% Triton-X 100. Subsequently, the cells were incubated with secondary antibodies ( Table 3) for four hours at RT. The coverslips were then washed two times five minutes in PBS followed by a five minute wash in PBS containing Hoechst (5 µg/µl, Sigma-Aldrich) before being mounted using Fluorescence Mounting Medium (Dako) and stored at 4°C. As negative controls of the immunostaining, simultaneous stainings were done using a similar protocol, except primary antibody was omitted.

### Confocal microscopy of hippocampal tissue, spinal cord tissue and cultured hippocampal neurons

- Confocal microscopy. The samples were analysed on a Zeiss confocal LSM 780 microscope (Carl Zeiss) using 20X/0.8 M27 and 63X/1.20 W Korr (Water immersion correction ring) objectives. Appropriate filters were used upon excitation of the different fluorophores to match their maximum fluorescence emission. The channels used were H258 and A568 and they were configured to obtain the best signal during image acquisition of the samples in order to prevent bleed through between the different probes. The range indicator was used to adjust gain and offset so acquired images were optimally held within the dynamic range of the detector. Frame size was selected to be “optimal” and an averaging of 16 was selected upon image acquisition in order to acquire an appropriate number of pixels and to achieve a maximum of signal-to-noise-ratio, respectively. Image acquisition was performed with foci adjusted with respect to the 568 nm fluorophores, as they were used to visualize the markers of interneurons; parvalbumin, calretinin, calbindin and somatostatin. Processing of the acquired images were performed in Zen 2011 (Carl Zeiss) Image Processing. All images presented were subjected to similar brightness and contrast adjustments.

**Table 1.** List of chemicals and reagents. The use of each chemical can be found in the materials and methods section. The products are listed in alphabetic order.

Reagent	Working Concentration	Manufacturer	Catalog number
**Bovine Serum Albumin (BSA)**	1% w/v in TBS or TB buffer	Sigma ^®^	A4503
**B-27 ^®^ Supplement**	1x	Gibco ^®^ by Life Technologies	17504-044
**Deoxyribonuclease 1 (DNAse1)**	0.01 mg/mL	Sigma ^®^	DN25
**DMEM**	1x	Lonza	BE12-604F/U1
**D-PBS**	1x	Gibco ^®^ by Life Technologies	14190-094
**Fetal bovine serum (FBS)**	1x	Gibco ^®^ by Life Technologies	10270-106
**Fluorescence Mounting** **Medium**	n/a	Dako	S3023
**Floxuridine +** **Uridine**	20 μM 20 μM	Sigma ^®^ Sigma ^®^	F0503 U3750
**Formaldehyde**	4%	Hounisen	1000.5000
**GlutaMAX ^TM^ Supplement**	2 mM	Gibco ^®^ by Life Technologies	35050-061
**Hoechst**	5 μg/μL	Sigma-Aldrich ^®^	861405
**IsoFlo ^®^ vet**	4% gas	Abbott	002185
**Iso-Pentane**	n/a	VWR BDH Prolabo ^®^	24872.298
**Laminin**	20 μg/mL	Invitrogen	23017-015
**Neurobasal-A ^®^ Medium**	n/a	Gibco ^®^ by Life Technologies	10888-022
**Pentobarbital 50 mg/mL**	5 mg/mL	The pharmacy at Aarhus University	
**Paraformaldehyde**	4% w/v in PBS, pH 7.4	Sigma Aldrich ^®^	P6148
**Papain**	20 U/mL	Worthington Biochemical Corporation	LS003126
**Poly-D-Lysine**	0.1 mg/mL	Sigma-Aldrich ^®^	P6407
**Primocin ^TM^**	100 μg/mL	Invivogen	ant-pm-2
**Sucrose**	30% w/v in PBS	Merck Millipore	1.07687.1000
**Target Retrieval Solution**	1x	Dako	S1699
**Tissue-Tek ^®^ O.C.T ^TM^ compound**	n/a	Sakura	4583
**Tris Base buffer (TB buffer)**	50 mM Tris Base	Calbiochem	648311
**Tris-buffered saline (TBS)**	50 mM Tris Base 150 mM NaCL	Calbiochem Merck Millipore	648311 1.06404.1000
**Triton ^®^ X-100**	0.3% in TBS for IHC 0.1% in PBS for ICC	Applichem	A1388

**Table 2.** Primary antibodies used for immunostaining of ^1^hippocampal sections, ^2^hippocampal neurons and ^3^spinal cord sections. The pAbmAbs rating reflects the average rating of the antibodies as of October 2014.

Antibody	Host	Clonality	Immunogen	Dilution factor	Company	Catalog nr. batch nr.	RRID	pAbmAbs rating (1–5)
**Anti-** **Calbindin ^1, 3^**	Rabbit	Polyclonal	Recombinant mouse calbindin	1:500	Millipore	Ab1778 2040376	AB_2068336	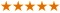
**Anti-** **Calbindin ^1, 2^**	Mouse	Monoclonal	Bovine kidney calbindin-D	1:500	Sigma- Aldrich ^®^	C9848 052M4833	AB_476894	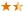
**Anti-** **Calbindin ^1, 2^**	Rabbit	Monoclonal	Chicken gut calbindin D-28k	1:200	Swant	D28K 07 (F)	n/a	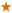
**Anti-** **Calretinin ^1, 3^**	Rabbit	Polyclonal	Recombinant rat calretinin	1:1000	Millipore	Ab5054 20 xx 170	AB_2068506	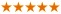
**Anti-** **Calretinin ^1, 2^**	Sheep	Polyclonal	Native guinea pig calretinin	1:500	Rockland	200-601-D13 28000	AB_11183443	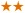
**Anti-** **Calretinin ^1, 2^**	Mouse	Monoclonal	Recombinant rat calretinin	1:1000	Millipore	Mab1568 2123143	AB_94259	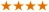
**Anti-** **Calretinin ^1, 2^**	Mouse	Monoclonal	Recombinant human calretinin	1:200	Swant	6B3 010399	AB_10000320	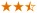
**Anti-** **Parvalbumin ^1– 3^**	Rabbit	Polyclonal	Rat parvalbumin	1:1000	Abcam	Ab11427 GR101095-2	AB_298032	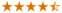
**Anti-** **Parvalbumin ^1, 2^**	Guinea pig	Polyclonal	Recombinant rat parvalbumin	1:250	Synaptic systems	195 004 195004/11	AB_2156476	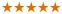
**Anti-** **Parvalbumin ^1, 2^**	Mouse	Monoclonal	Frog muscle parvalbumin	1:2000	Sigma- Aldrich ^®^	P3088 100M4797	AB_477329	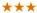
**Anti-** **Parvalbumin ^1, 2^**	Rabbit	Polyclonal	Synthetic peptide	1:250	Millipore	Ab15736 1869268	AB_838238	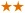
**Anti-** **Somatostatin ^1– 3^**	Rat	Monoclonal	Synthetic peptide	1:1000	Millipore	Mab354 2060939	AB_2255365	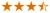
**Anti-** **Somatostatin ^1, 2^**	Rabbit	Polyclonal	Synthetic human peptide	1:250	Sigma- Aldrich ^®^	SAB4502861 310328	AB_10747468	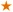

**Table 3.** Secondary antibodies used for immunostaining of ^1^hippocampal sections, ^2^hippocampal neurons and ^3^spinal cord sections.

Antibody	Host	Fluorescent dye	Dilution factor	Company	Catalog nr.
**α-Rabbit IgG (H+L) ^1– 3^**	Donkey	Alexa Fluor ^®^ 568	1:300	Molecular probes ^®^	A-10042
**α-Mouse IgG (H+L) ^1, 2^**	Donkey	Alexa Fluor ^®^ 568	1:300	Molecular probes ^®^	A-10037
**α-Sheep IgG (H+L) ^1, 2^**	Donkey	Alexa Fluor ^®^ 568	1:300	Molecular probes ^®^	A-21099
**α-Guinea Pig IgG (H+L) ^1, 2^**	Donkey	CF ^TM^ 488A	1:300	Sigma	SAB4600033
**α-Rat IgG (H+L) ^1, 2^**	Goat	Alexa Fluor ^®^ 568	1:300	Molecular probes ^®^	A-11077
**α-Rat IgG (H+L) ^3^**	Donkey	Alexa Fluor ^®^ 594	1:300	Molecular probes ^®^	A-21209

## Results and discussion

### Interneurons of the hippocampus

Initially, we screened the antibody specificity by staining of cultured hippocampal neurons, evaluating antibodies based on their ability to mark a distinct subset of neurons. Hereafter, when staining hippocampal sections, the antibodies were rated based on the expected localization and abundance of interneurons positive for the specific staining.

**Figure 1.**
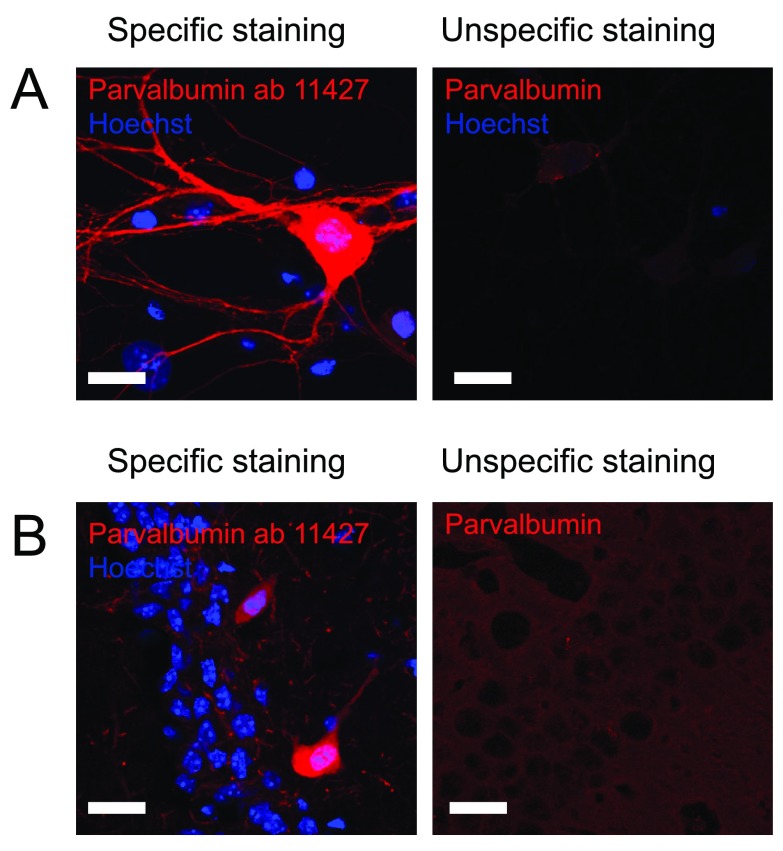
Staining against parvalbumin interneurons. Figure 1 shows immunostaining against parvalbumin on **A**) cultured hippocampal neurons and **B**) hippocampal tissue. Left pictures shows an example of an immunostaining considered to be specific while right picture shows an example where immunostaining using other primary antibodies did not meet the criteria and therefore was considered unspecific. Scale bar represents 20 µm.

The localization of parvalbumin interneurons within the dentate gyrus is very well described so cells staining positive in layers where parvalbumin interneurons are not expected were considered as unspecific immunostaining. For several of the immunostainings, very little, if any, signal was obtained. However, the anti-parvalbumin ab11427 antibody from Abcam gave a clear and intense staining of parvalbumin interneurons both in culture and in hippocampal tissue sections ( Figure 1 and Table 2). As the positive neurons were found in layers of the dentate gyrus, where parvalbumin positive interneurons have previously been described to be located, at an expected frequency, this was considered a specific staining and was therefore rated with 5 out of 5 stars on pAbmAbs ( www.pAbmAbs.com).

**Figure 2.**
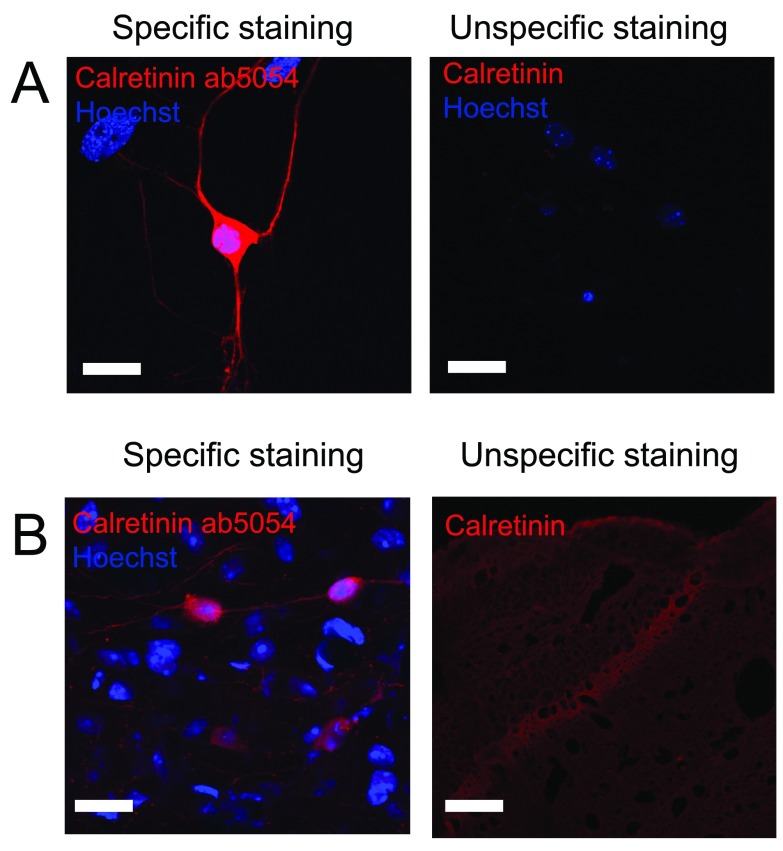
Staining against calretinin. Figure 2 shows immunostaining against calretinin on **A**) cultured hippocampal neurons and **B**) hippocampal tissue. Left pictures shows an example of an immunostaining considered to be specific while right picture shows an example where immunostaining using other primary antibodies did not meet the criteria and therefore was considered unspecific. Scale bar represents 20 µm.

Unlike parvalbumin, calretinin is found not only in interneurons but also in mossy cells within the dentate gyrus. These can often be distinguished based on the intensity of the labelling. When rating these antibodies, the correct localization of positive neurons was therefore considered not only in relation to interneurons but also to mossy cells. Both antibodies from Millipore showed high specificity against calretinin, and especially the anti-calretinin ab5054 antibody gave a very specific staining with a high signal-to-noise ratio and was therefore given 5 out of 5 stars on pAbmAbs ( Figure 2 and Table 2).

**Figure 3.**
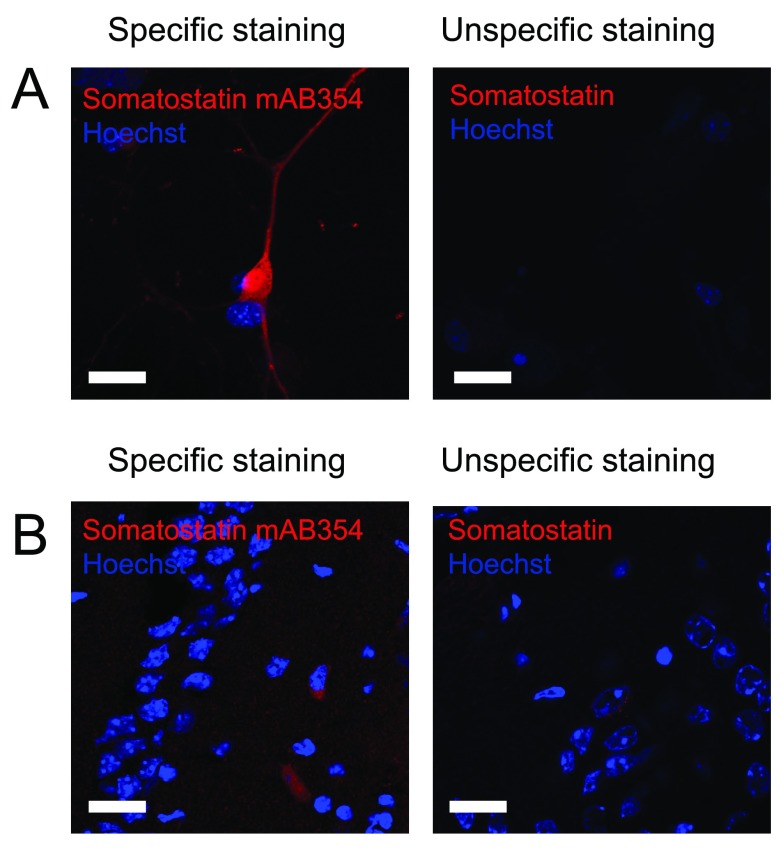
Staining against somatostatin. Figure 3 shows immunostaining against somatostatin on **A**) cultured hippocampal neurons and **B**) hippocampal tissue. Left pictures shows an example of an immunostaining considered to be specific while right picture shows an example where immunostaining using other primary antibodies did not meet the criteria and therefore was considered unspecific. Scale bar represents 20 µm.

Similarly, antibodies against somatostatin were evaluated based on signal-to-noise and localization of neurons positive for somatostatin. In most cases, staining against somatostatin gave a high background with very low signal. However, using the anti-somatostatin mab364 antibody from Millipore we observed a clear staining with a good signal-to-noise ratio ( Figure 3 and Table 2) and therefore it received a rating of 5 out of 5 stars. The neurons positive for somatostatin were, as expected, found in the hilus of the dentate gyrus.

**Figure 4.**
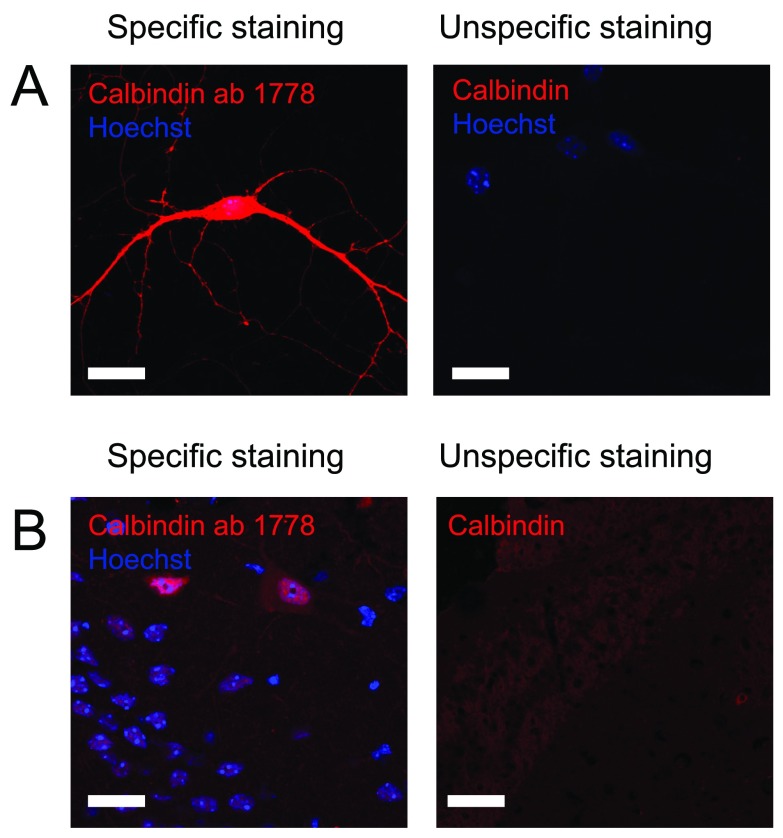
Staining against calbindin. Figure 4 shows immunostaining against calbindin on **A**) cultured hippocampal neurons and **B**) hippocampal tissue. Left pictures shows an example of an immunostaining considered to be specific while right picture shows an example where immunostaining using other primary antibodies did not meet the criteria and therefore was considered unspecific. Scale bar represents 20 µm.

Like calretinin, calbindin is also expressed by non-inhibitory cells. When looking at the dentate gyrus, expression of calbindin by principal cells within the granule cell layer gives a weak immunostaining which might seem like unspecific binding, however that is not the case. Interneurons positive for calbindin can be recognized based on the location as well as increased intensity of the immunostaining. Due to the very low number of calbindin-interneurons in the hilus, this immunostaining can be hard to detect. Many of the antibodies we tested showed very little if any difference in staining intensity between interneurons and granule cells. However, using the anti-calbindin ab1778 antibody from Millipore we were able to distinguish between interneurons and granule cells ( Figure 4 and Table 2). Since this antibody also shows very little background staining it was rated 5 stars on pAbmAbs.

### Interneurons of the spinal cord

Parvalbumin positive cells of the spinal cord dorsal horn also represent a subgroup of GABAergic interneurons and immunostaining against parvalbumin can accordingly be used as a marker of GABAergic interneurons. When staining against parvalbumin with the anti-parvalbumin ab11427 antibody from Abcam they appeared to be largely restricted to laminae II-III of the dorsal horn, which is in accordance with previous findings ^27^. The parvalbumin positive cells of laminae II-III were rather small and showed intense immunoreactivity in the nucleus and in the soma, as previously described ^22^, making it easy to distinguish them from background staining. This antibody also appeared to stain neuronal processes of the dorsal horn and columns as well as the nuclei of ventral horn motor neurons, as previously described ^27– 29^. Although this antibody can be used to identify intense immunoreactive parvalbumin positive cells and function as a great marker of the parvalbumin positive subpopulation of GABAergic neurons of the spinal dorsal horn in locations previously described, it showed some background staining of spinal cord cryo-sections and was rated 4 out of 5 stars on pAbmAbs.

Unlike interneurons of the hippocampus, calretinin can only be used as a marker of interneurons that do not contain GABA in the spinal cord ^24^. The anti-calretinin AB5054 antibody from Merck Millipore works well for IHC of spinal cord cryo-sections (data not shown) and was rated 5 out of 5 stars on pAbmAbs, as it showed very low background staining and intense staining of a dense well-defined band of small calretinin immunoreactive cells in the superficial laminae of the dorsal horn and of large cells in lamina V-VI. These observations correlates with previous description of calretinin immunoreactivity in the spinal cord ^24^, and indicates high specificity of the antibody.

In contrast to IHC of hippocampal sections with the anti-somatostatin MAB354 antibody from Millipore, this antibody gave a low signal when staining against somatostatin on spinal cord sections. Using this antibody, it was difficult to identify somatostatin positive cells in the spinal dorsal horn that otherwise previously have been described to be located in a dense band in lamina II of rat ^25^ and mouse ^21^ spinal dorsal horn. Therefore, the antibody was rated 2 out of 5 stars on pAbmAbs. This antibody was rated 5 out of 5 for the hippocampal staining, leading to an average rating of 3.5 on pAbmAbs.

Like calretinin and somatostatin, calbindin can be used as a marker of spinal dorsal horn interneurons that do not contain GABA ^23^. A dense band of calbindin immunoreactivity has previously been shown in lamina II and a more sparse band in lamina I, III and IV of the rat spinal dorsal horn ^23^. This localization of calbindin immunoreactivity is also seen when using the anti-calbindin AB1778 antibody from Merck Millipore (data not shown). Also, the cells that constitute the central channel and motor neurons of the ventral horn also show calbindin immunoreactivity when staining with this antibody, which is in accordance with previously findings ^28, 30^. The antibody showed very intense staining of cytoplasm and nuclei, as well as processes of the outer lamina of the dorsal horn and showed low background staining. On the basis of these observations the antibody was rated 5 out of 5 stars on pAbmAbs.

Dataset 1. Interneurons of hippocampus and spinal cord http://dx.doi.org/10.5256/f1000research.5349.d36682
The raw microscopy images for both hippocampal and spinal cord interneurons are shown in the .czi files provided.

## Conclusion

In conclusion, staining against interneurons can be a very tedious task and great consideration is needed to ensure that it is actually only interneurons that are being stained. Optimizing protocols for immunostaining can be a, not only time consuming, but also an expensive task in a market full of different antibody options. By creating an information-sharing platform, pAbmAbs allows for a fast and cost-free screening of the current antibodies out there and thereby ensures that only the best antibodies are used. In the current study, we tested antibodies against parvalbumin, calretinin, calbindin and somatostatin, all markers of hippocampal GABAergic interneurons, both in culture and on hippocampal and spinal cord tissue. These antibodies were rated on specificity, and signal-to-noise ratio, for both tissue and culture. When immunostaining tissue, we also looked at the localization of positive cells within the tissue to ensure that only cells in the expected layers of the tissue stained positive for the GABAergic markers. When staining against parvalbumin we found that out of four different antibodies, the anti-parvalbumin ab11427 antibody from Abcam got a high score as it stained cells specifically with a high signal-to-noise ratio with the expected localization within the tissue. When staining against calretinin, the anti-calretinin ab5054 antibody from Millipore obtained the highest score on pAbmAbs. This antibody gave a very nice signal-to-noise ratio compared to the other antibodies tested. The anti-somatostatin mab354 antibody from Millipore was found to be the best antibody for stainings against somatostatin. Similar to the other antibodies with high pAbmAbs ratings, this also had a high signal-to-noise ratio compared to other antibodies tested. Finally, the anti-calbindin ab1178 antibody from Millipore obtained the highest rating out of the antibodies tested against this GABAergic subgroup. Overall, the antibody tested gave varying results when using our protocols. The specificities of the antibodies are therefore reflected on pAbmAbs which, by serving as a database, will help fast and cost-free evaluation of antibodies.

## Data availability

*F1000Research*: Dataset 1. Interneurons of hippocampus and spinal cord, 10.5256/f1000research.5349.d36682 ^31^
