# Supplementary material for: Immunofluorescent visualization of mouse interneuron subtypes
Source: F1000Res. 2014 Nov 20;3:242. Originally published 2014 Oct 13. [Version 2] doi: 10.12688/f1000research.5349.2 (PMC4240249; doi:10.12688/f1000research.5349.2)
Supplement: Peer review history

# Peer review: Referee response for version 1

**DOI:** 10.5256/f1000research.5710.r6402

**Published:** 2014-10-21

**Authors:** Mei Yee Leung

**Affiliations:** 1 St John's Laboratory Ltd., London, UK

This is an informative, concise article with clear aims that highlights the potential difficulties in selecting the right antibodies for specific cell types and research applications. In this study, the authors systematically tested commercial antibodies against calbindin, calretinin, parvalbumin and somatostatin - markers of GABAergic interneuron subtypes. Of the 13 antibodies tested, only 4 were deemed reliable and useful for characterizing these subtypes in mouse brain.

The authors’ rigorous approach in selecting the right antibodies is commendable, a fact which often goes unnoticed in publications. The ranking of antibody performance in pAbmAbs, a review-based platform is a much needed resource for scientists whose research depends on the validity of the antibodies.

As someone not in this research area, I found the manuscript scientifically well construed and the rationale easy to follow. It is noteworthy that this study by Molgaard *et al.* adds to the understanding of GABAergic subtypes in the mouse hippocampus and spinal cord, information which is sparse in the literature. In addition, by demonstrating specific staining of these markers, they have confirmed previously reported localization of these cells.

A few comments/suggestions

- Would a change in the title be more appropriate? e.g.  Immunofluorescent instead of immunohistochemical
- Is there any reason why the age of the mouse used for hippocampal staining and that for spinal cord staining is different?
- It would add value to this paper is images of spinal cord staining was also shown
- Although the without primary images are very clean,  using these antibodies on tissues not known to express these targets would be a more superior negative control
- Could the authors offer an explanation for why the polyclonal antibodies appear to perform better than the monoclonals?
- To obtain better idea of reproducibility, it would be good to give an indication of how many times the experiment was performed and how many sections were stained per experiment

I have read this submission. I believe that I have an appropriate level of expertise to confirm that it is of an acceptable scientific standard.

# Response

**Published:** 2014-11-12

**Authors:** Simon Molgaard

**Affiliations:** Aarhus University, Denmark

*"Would a change in the title be more appropriate? e.g.  Immunofluorescent instead of immunohistochemical"*

**Reply**: OK

*"Is there any reason why the age of the mouse used for hippocampal staining and that for spinal cord staining is different?"*

**Reply**: No particular reason. The expression of GABAergic markers in the hippocampus and spinal cord of adult animals is expected to be fairly constant throughout adulthood.

*"It would add value to this paper is images of spinal cord staining was also shown"*

**Reply**: Done

*"Although the without primary images are very clean, using these antibodies on tissues not known to express these targets would be a more superior negative control"*

**Reply**: This point may rely on a possible misunderstanding. The images in the paper denoted “Unspecific staining” refers to stainings using primary antibodies for which no specific signal was observed. We have changed the legends to make this more clear.

*"Could the authors offer an explanation for why the polyclonal antibodies appear to perform better than the monoclonals?"*

**Reply**: We have no clear answer to this but it may be that polyclonal antibodies generally give a higher signal compared monoclonal antibodies due to the presence of multiple epitopes.

*"To obtain better idea of reproducibility, it would be good to give an indication of how many times the experiment was performed and how many sections were stained per experiment"*

**Reply**: This is now stated in the Methods section.

# Peer review: Referee response for version 1

**DOI:** 10.5256/f1000research.5710.r6403

**Published:** 2014-10-17

**Authors:** Tomi PJ Rantamäki

**Affiliations:** 1 Neuroscience Center, University of Helsinki, Helsinki, Finland

Molgaard *et al.* have investigated the suitability of various commercially available antibodies for the identification of GABAergic interneurons in mice. Among 13 tested antibodies against calbindin (3), calretinin (4), parvalbumin (4) and somatostatin (2), the authors found 1-2 antibodies per each marker that produced high quality, sensitive and specific staining in mouse brain sections and cultured hippocampal neurons obtained from P0 mouse pups. Their findings remind us about the tediousness of immunostainings in general and challenges about the identification of proper antibody and antibody conditions suitable for high quality research. Although the antibodies have not been investigated in all possible experimental conditions (e.g. fixation protocols, dilutions), the study provides very valuable reference information for researchers aiming to investigate GABAergic markers in mice.          

In general the manuscript has been written and constructed well. Abstract nicely describes the summary of the study. The introduction provides very good background knowledge for the reader (incl. relevant citations). The methods section is described in a manner that allows scientific reproduction efficiently. Tables are clear and useful. Overall the representative figures are good but the paper would benefit with more comprehensive set of immunostainings (as main figures).  

I have few minor comments/questions:

- GABAergic interneurons are considered as small neuronal population in the text. In respect to glutamatergic neurons this is indeed the case, but overall I consider 20% quite a significant fraction (cf. monoaminergic neurons).
- The authors could have clarified what is “pAbmAbs” in the abstract.
- In the Introduction the authors state that breakdown of cortical network rhythms underlie neuropsychiatric disorders. I would rather say that alterations in cortical network rhythms in specific brain networks may underlie neuropsychiatric disorders.
- The authors could have explained that glutamate decarboxylase is expressed in GABAergic neurons and it synthesizes GABA (Introduction, first and second paragraph).
- The authors could have described the gender and amount of adult mice used for the study. Moreover, where all the antibodies tested in specimens derived from same conditions (e.g. same animal).
- I would have used “sections” rather than “slices” throughout the paper.
- Table 2. would be even more clear if the antibodies against the four different markers would have been divided from each other more clearly (e.g. using different background colors).
- Why there appears no Hoechst staining in some of the unspecific stainings?
- Why the authors choose not to show the representative figures from spinal cord?
- The authors could have emphasized that the quality of polyclonal antibodies is significantly determined by the lot/batch. This should be kept in mind when reproducing the findings.
- Figure legends should have been clearer. In optimal case, the reader understands the figures thoroughly without the main text (e.g. age of cultures).
- It would have been very useful to test, at least selected, antibodies in rats as well (brain sections, culture) 

I have read this submission. I believe that I have an appropriate level of expertise to confirm that it is of an acceptable scientific standard.

# Response

**Published:** 2014-11-12

**Authors:** Simon Molgaard

**Affiliations:** Aarhus University, Denmark

*"GABAergic interneurons are considered as small neuronal population in the text. In respect to glutamatergic neurons this is indeed the case, but overall I consider 20% quite a significant fraction (cf. monoaminergic neurons)."*

**Reply**: Comment well taken. The word “small” has now been removed from the abstract.

*"The authors could have clarified what is “pAbmAbs” in the abstract."*

**Reply**: This has been added to abstract.

*"In the Introduction the authors state that breakdown of cortical network rhythms underlie neuropsychiatric disorders. I would rather say that alterations in cortical network rhythms in specific brain networks may underlie neuropsychiatric disorders."*

**Reply**: We agree. The text is now changed accordingly.

*"The authors could have explained that glutamate decarboxylase is expressed in GABAergic neurons and it synthesizes GABA (Introduction, first and second paragraph)."*

**Reply**: This has now been clarified in the introduction.

*"The authors could have described the gender and amount of adult mice used for the study. Moreover, where all the antibodies tested in specimens derived from same conditions (e.g. same animal)."*

**Reply**: A paragraph has been added to the Methods clarifying these issues.

*"I would have used “sections” rather than “slices” throughout the paper."*

**Reply**: This has now been corrected.

*"Table 2. would be even more clear if the antibodies against the four different markers would have been divided from each other more clearly (e.g. using different background colors)."*

**Reply**: Although we agree that a color-code would be desirable, we feel that the current table is sufficiently clear.

*"Why there appears no Hoechst staining in some of the unspecific stainings?"*

**Reply:** We apologize for the lack of clarity regarding this issue. The “unspecific” images with Hoechst are shown when we observed no signal at all. The “unspecific” images without Hoechst are shown when staining was present but distributed in an unspecific manner.

*"Why the authors choose not to show the representative figures from spinal cord?"*

**Reply**: The images are already included in the data availability section.

*"The authors could have emphasized that the quality of polyclonal antibodies is significantly determined by the lot/batch. This should be kept in mind when reproducing the findings."*

**Reply**: Good point. In this regard we will refer to the article in the antibody validation collection by Dr Jan Voskuil. 

*"Figure legends should have been clearer. In optimal case, the reader understands the figures thoroughly without the main text (e.g. age of cultures)."*

**Reply**:  The figure legends have now been improved

*"It would have been very useful to test, at least selected, antibodies in rats as well (brain sections, culture) "*

**Reply**: We agree but as stated in the title we have only used mouse.

# Peer review: Referee response for version 1

**DOI:** 10.5256/f1000research.5710.r6401

**Published:** 2014-10-14

**Authors:** Sally Lowell

**Affiliations:** 1 MRC Centre for Regenerative Medicine, Institute for Stem Cell Research, School of Biological Sciences, University of Edinburgh, Edinburgh, UK

The authors of this paper set out to identify antibodies that can be used to identify particular subtypes of GABAergic neurons within the mouse hippocampus. They explain that many of the antibodies that they have tested for this purpose do not seem to work, and they present data for those antibodies that they did find to be useful for this purpose. This article could therefore save readers a lot of wasted effort and money in identifying useful antibodies for their own studies in this area.

The article starts with a nice overview of the different subtypes of GABAergic neurons and the markers that are commonly used to characterise them. I am not an expert in this area so I cannot review the accuracy of the information, but I did find it to be concise and useful introduction.

 There are a few issues that could be addressed to improve the results section of the article.

1. Figure legends should explain what "unspecific staining" refers to. Does this mean secondary only control? If so then this form of labelling could be misleading as non-specific binding of the primary antibody will not be picked up by a secondary-antibody-only control.
2. Why are there no Hoechst positive cells in  some of the 'unspecific staining' panels?
3. The evidence that these antibodies are specific to particular subtypes of neurons comes mainly from the observation that their staining pattern is restricted to the expected regions of the hippocampus. However the figures as presented do not make this clear. Is it possible to provide a clearer demonstration of these restricted expression domains on hippocampus sections? Perhaps it would be helpful to see an accompanying diagram showing the expected distribution of each marker on these sections?
4. In Fig 3B, the 'specific staining' of antibody mAB354 is barely visible so it is difficult to assess whether there is a real difference between signal and background.
5. The article reports results from immunostaining of the spinal cord, but no figures are presented to support the authors’ findings.  It would be helpful to see these images.

General comments:  The article is written clearly and concisely, but would benefit from being proof-read for minor grammatical errors.

I have read this submission. I believe that I have an appropriate level of expertise to confirm that it is of an acceptable scientific standard, however I have significant reservations, as outlined above.

# Response

**Published:** 2014-11-12

**Authors:** Simon Molgaard

**Affiliations:** Aarhus University, Denmark

*"Figure legends should explain what "unspecific staining" refers to. Does this mean secondary only control? If so then this form of labelling could be misleading as non-specific binding of the primary antibody will not be picked up by a secondary-antibody-only control."*

**Reply**: The figure legends have been changed to make this more clear.

*"Why are there no Hoechst positive cells in some of the 'unspecific staining' panels?"*

**Reply**: We apologize for the lack of clarity regarding this issue. The “unspecific” images with Hoechst are shown when we observed no signal at all. The “unspecific” images without Hoechst are shown when staining was present but distributed in an unspecific manner.

*"The evidence that these antibodies are specific to particular subtypes of neurons comes mainly from the observation that their staining pattern is restricted to the expected regions of the hippocampus. However the figures as presented do not make this clear. Is it possible to provide a clearer demonstration of these restricted expression domains on hippocampus sections? Perhaps it would be helpful to see an accompanying diagram showing the expected distribution of each marker on these sections?"*

**Reply**: The interneuron subtype distribution is presently used in a qualitative manner to validate whether positive staining localizes in the expected and with an expected frequency in the dentate gyrus of the hippocampus. Quantitative assessment of interneuron number using for example stereology is not feasible in the present study.

*"In Fig 3B, the 'specific staining' of antibody mAB354 is barely visible so it is difficult to assess whether there is a real difference between signal and background."*

**Reply**: We agree that the staining is weaker; however it is still distinct and specific.

*"The article reports results from immunostaining of the spinal cord, but no figures are presented to support the authors’ findings.  It would be helpful to see these images."*

**Reply**: The images are already included in the data availability section.
